# Understanding non-response to cardiac resynchronisation therapy: common problems and potential solutions

**DOI:** 10.1007/s10741-018-9734-8

**Published:** 2018-08-24

**Authors:** Benjamin J. Sieniewicz, Justin Gould, Bradley Porter, Baldeep S. Sidhu, Thomas Teall, Jessica Webb, Gerarld Carr-White, Christopher A. Rinaldi

**Affiliations:** 1 Division of Imaging Sciences and Biomedical Engineering, King’s College London, 4th Floor, North Wing, St Thomas’ Hospital, London, SE1 7EH UK; 2 Cardiology Department, Guys and St Thomas’ NHS Foundation Trust, London, SE1 7EH UK

**Keywords:** CRT, Heart failure, Non-responders, Endocardial pacing

## Abstract

Heart failure is a complex clinical syndrome associated with a significant morbidity and mortality burden. Reductions in left ventricular (LV) function trigger adaptive mechanisms, leading to structural changes within the LV and the potential development of dyssynchronous ventricular activation. This is the substrate targeted during cardiac resynchronisation therapy (CRT); however, around 30–50% of patients do not experience benefit from this treatment. Non-response occurs as a result of pre-implant, peri-implant and post implant factors but the technical constraints of traditional, transvenous epicardial CRT mean they can be challenging to overcome. In an effort to improve response, novel alternative methods of CRT delivery have been developed and of these endocardial pacing, where the LV is stimulated from inside the LV cavity, appears the most promising.

## Introduction

Heart failure is a complex clinical syndrome associated with a significant morbidity and mortality burden. Reductions in left ventricular (LV) function trigger adaptive mechanisms aimed at regulating the cardiac output; however, over time these processes cause structural changes within the LV. This remodelling can result in the development of dyssynchronous ventricular activation, typically manifested by left bundle branch block on the 12-lead ECG. This is the substrate targeted during cardiac resynchronisation therapy; however, between 30 and 50% of patients do not experience significant benefits from this treatment [1]. This review will explore the reasons why some patients fail to respond to traditional transvenous, epicardial CRT and how novel methods of pacing may offer a potentially better strategy.

## Ventricular remodelling

In response to the progressive haemodynamic stress associated with heart failure, compensatory structural changes occur within the heart. The left ventricular myocardial mass and composition exhibit evidence of change. As this occurs, the heart becomes less elliptical and more spherical as the geometry and volume of the LV change. This process is called remodelling and initially enables the heart to increase cardiac output by increasing both contractility and stroke volume. Whilst stroke volume initially increases, this process eventually becomes deleterious. Progressive enlargement of the ventricle leads to hypertrophy, causing increased wall tension and ultimately results in the myocardium becoming fibrotic, impairing contractility. As the left ventricle dilates and remodels, it exhibits greater contractile dyssynchrony further reducing efficiency.

## Electrical dyssynchrony

Dyssynchronous electrical conduction can manifest as abnormal heart rate modulation, disruption to the sequence of atrio-ventricular systole and overt ventricular systolic discoordination which typically manifests as bundle branch block on the 12-lead ECG. The development of left bundle branch block is thought to occur as a result of ventricular remodelling and/or fibrogenic damage to the cardiac condition system [2]. Progressive increases in the QRS duration herald deterioration in LV performance [3–6]. Left bundle branch block results in dyssynchronous ventricular activation causing an abnormal pattern of mechanical activation and contraction.

In the context of left bundle branch block, the right anterior septal region is initially activated via the intact right bundle in juxtaposition to the left basal posterolateral region, which slowly propagates via cell-to-cell, intra-myocardial conduction. Contraction of the anterior right septum is imbalanced due to delayed activation of the lateral free wall and occurs unopposed. This has the effect of delaying elevation in the intra-cavity pressure gradient (dP/dtmax) as septal activation merely results in pre-stretch of the inactive lateral wall. When the lateral wall finally contracts, the septum is now in diastole, generating an energy sink which further reduces the overall ejection of blood via the left ventricular outflow tract. Cardiac mechano-energetic efficiency is further exacerbated by delayed activation of the posterolateral papillary muscle, causing sub-optimal closure of the mitral valve and ultimately mitral regurgitation.

Over time, left bundle branch block (LBBB) activation causes molecular and cellular remodelling leading to alterations in glucose uptake, regional perfusion and calcium transport [7]. These factors can be pro-arrhythmic [8] and may help explain why patients with LBBB and chronic heart failure occasionally develop acute decompensation without a clear precipitant.

## The role of CRT in heart failure management

**Fig. 1:**
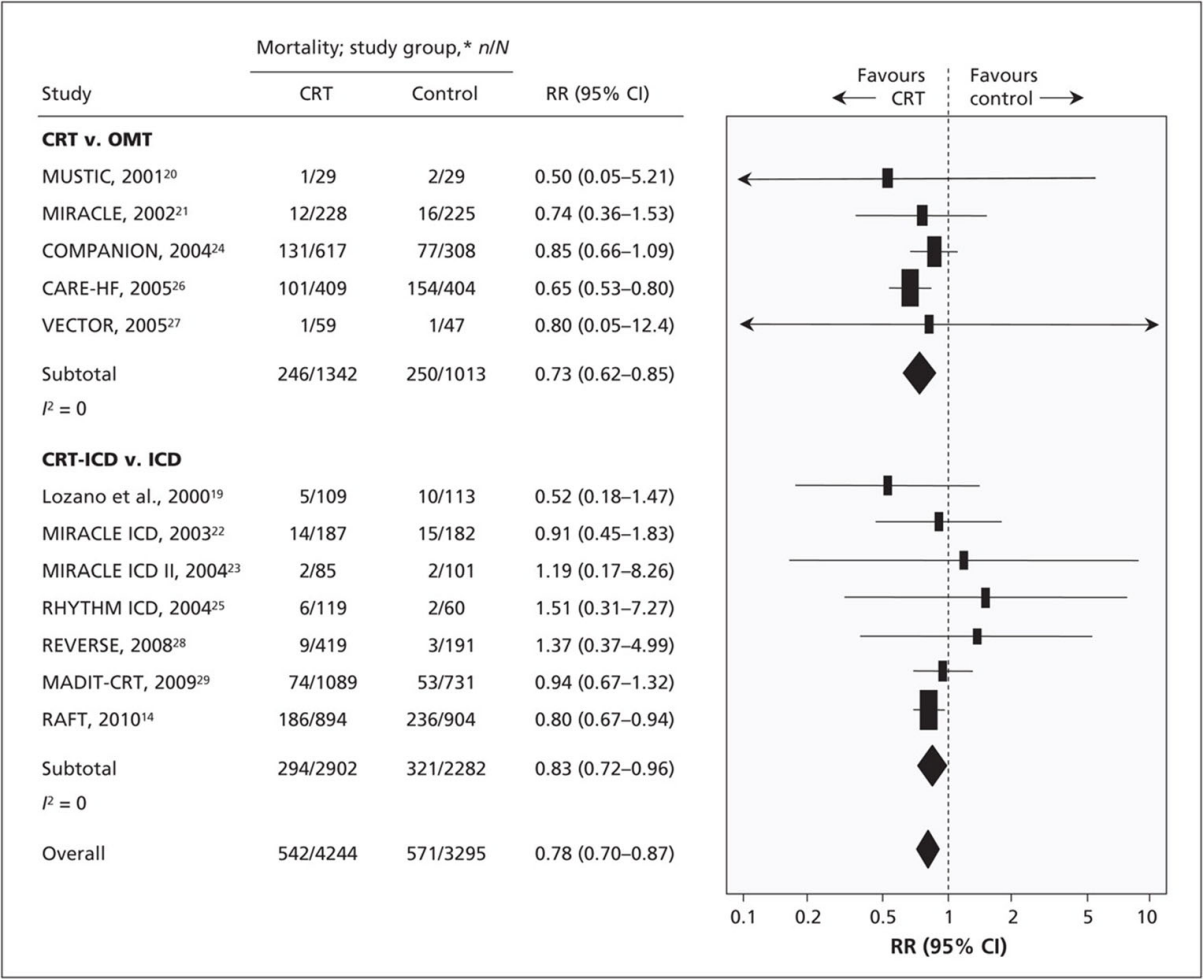
Results of random-effects meta-analysis of overall mortality amongst patients with heart failure given cardiac resynchronization therapy plus an implantable cardioverter defibrillator (CRT-ICD) versus an implantable defibrillator (ICD), by New York Heart Association (NYHA) class. Values less than 1.0 indicate a decreased risk of death with cardiac resynchronization therapy. Note CI = confidence interval, RR = relative risk. Reprinted from [9] © Canadian Medical Association (2011). This work is protected by copyright and the making of this copy was with the permission of the Canadian Medical Association Journal (www.cmaj.ca) and Access Copyright. Any alteration of its content or further copying in any form whatsoever is strictly prohibited unless otherwise permitted by law

Cardiac resynchronisation therapy (CRT) aims to eliminate the dyssynchrony which results from bundle branch block activation and restore the mechano-energetic efficiency of the heart. During CRT, both the left and right ventricles are stimulated in an attempt to re-coordinate cardiac electrical activation and produce a synchronous and efficient contraction. Several large randomised controlled trials of biventricular (BiV) pacing have been conducted which have established the efficacy of this therapy, which yields both reductions in morbidity and mortality, see Fig. 1 [9]. As a result of landmark studies, class 1 indications exist for CRT in both European [10] and American guidelines [11] in patients with symptomatic heart failure, a severely impaired LV (left ventricular ejection fraction, LVEF <35%) and an ECG demonstrating left bundle branch block with significant electrical delay.

### Response to CRT

Around 30–50% of patients fail to respond to transvenous, epicardial CRT. This group are classified as non-responders although no unifying definition of response to CRT exists. Response can be measured in a variety of different clinical, functional and structural endpoints and patients can fail to respond for a variety of different reasons.

**Fig. 2:**
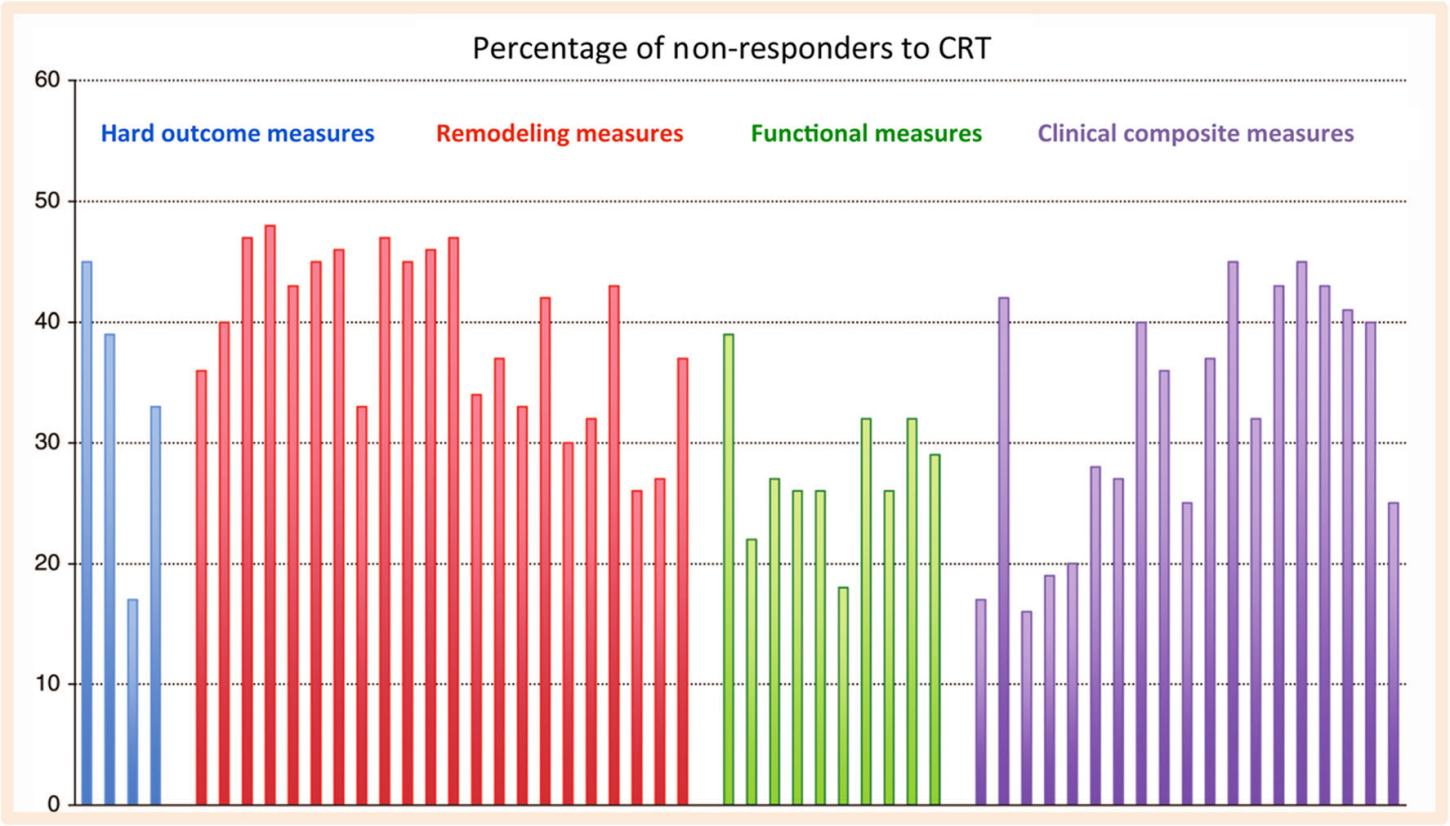
Rates of non-response to cardiac resynchronization therapy depending on the measure used in controlled trials and large observational studies of cardiac resynchronization therapy, each represented by a bar. Event-based measures are shown as blue, remodelling measures as red, functional and quality of life measures as green, and composite endpoints as purple bars. Reproduced with permission from [14]. Permission granted by Oxford University Press

The process of defining response to CRT is challenging as no universally accepted definition of CRT response exists. Response rates tend to be higher when clinical measures, such as subjective assessments of symptoms are used but are much lower when remodelling or outcome measurements are employed, see Fig. 2.

Additionally, symptomatic improvements do not always correlate with echocardiographic or functional classification improvements. There is also no consensus as to the optimal timeframe to re-assess LV function when gauging remodelling. The most widely accepted definition of response involves an assessment of left ventricular reverse remodelling (LVRR) 6 months after implantation, with reductions in LV end-systolic volume (LVESV) the most useful measure [12, 13]. Remodelling endpoints are typically associated with non-responder rates of between 30 and 45% [14] although in the systematic review published by Birnie and Tang (2006), the true figure appears somewhat higher at around 40–50% [15].

## Non-response to epicardial CRT

**Fig. 3:**
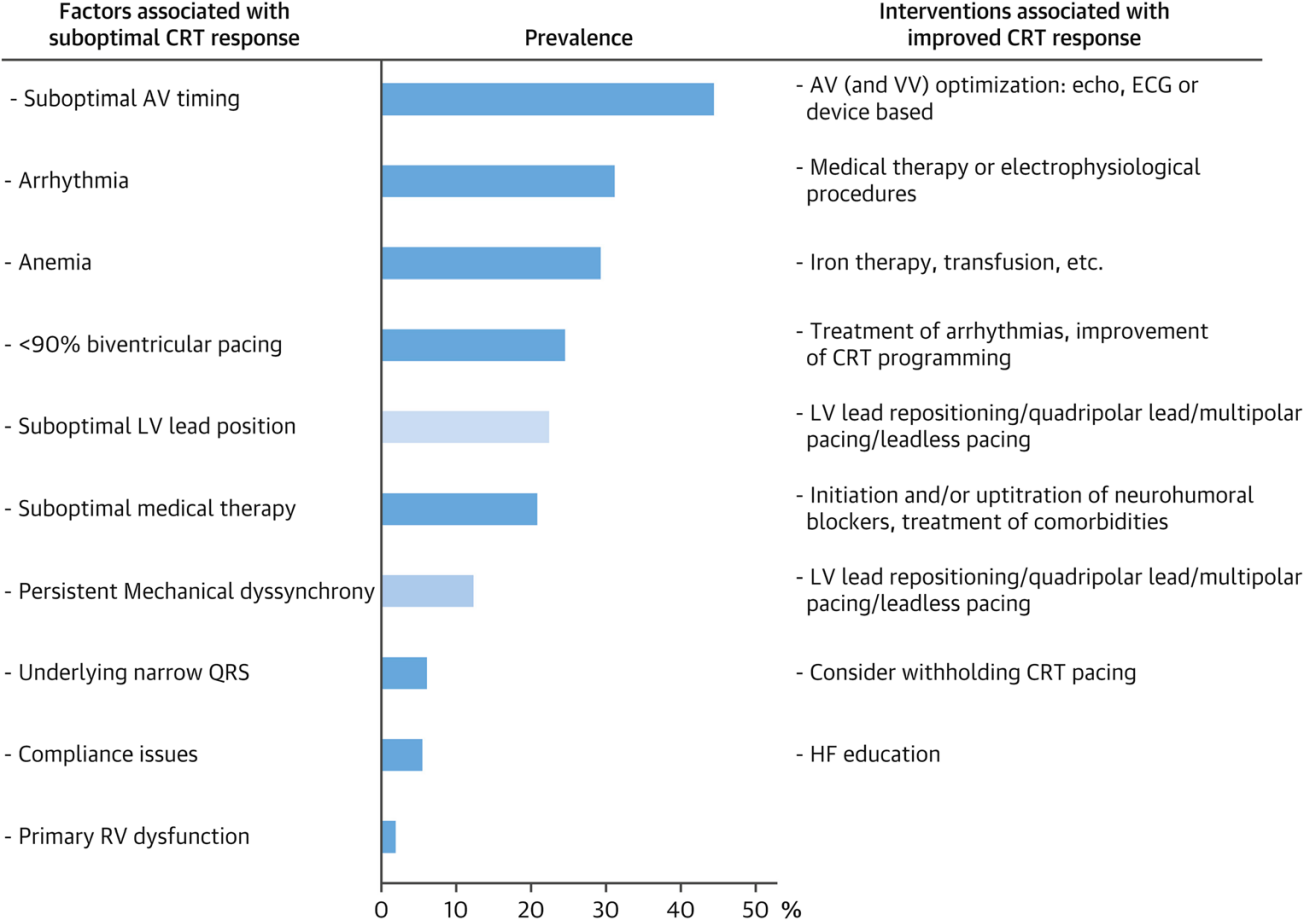
Factors associated with sub-optimal CRT response. Reproduced with permission from Wilfried Mullens and Petra Nijst. Understanding non-response to cardiac resynchronisation therapy; common problems and potential solutions. Journal of the American College of Cardiology. 2017 69(17):2130–2133

The reasons why some patients fail to adequately respond to transvenous, epicardial CRT are multifactorial and involve several pre-, peri- and post implant factors, see Fig. 3.

### Pre-implant

Pre-implant factors associated with non-response to CRT include appropriate patient selection, baseline ECG morphology and QRS duration, gender, aetiology, presence of myocardial scar and disease severity.

#### Patient selection

Optimal patient selection is critical when looking to maximise response to CRT. In both European [9] and American [10] guidelines, CRT is indicated in patients with symptomatic heart failure who exhibit impaired LV function and display evidence of ventricular dyssynchrony. Patients must have been established on optimal tolerated medical therapy and reversible causes of heart failure should have been excluded, including ischaemia, arrhythmia or valvular heart disease. In addition, current patient selection criteria utilise the surface 12-lead ECG to identify electromechanical delay; although recent sub-group analysis has suggested that the actual pattern of activation may in fact be more important determinant than the actual QRS width [16, 17].

#### ECG morphology and QRS duration

The presence of left bundle branch block morphology is a strong predictor of response to CRT [18]. Whilst no definitive data exists evaluating CRT response in patients with right bundle branch block (RBBB), retrospective analysis suggests that this group of patients tends to do less well. Interestingly, when patients with heart failure and RBBB were analysed using 3D non-contact mapping, they were found to exhibit significant LV activation delay in addition to the delay identified in the RV [19]. In addition, LBBB activation is not exclusively associated with electrical conduction delay [20]. In one analysis, up to a third of patients with LBBB who underwent electromechanical or non-contact mapping demonstrated normal trans-septal activation time and a near-normal LV endocardial activation time [21]. It is possible that more nuanced assessments of electrical delay, such as non-invasive body surface mapping, may be able to detect remediable patterns of electrical delay with greater accuracy.

The evidence for CRT in patients with non-specific intraventricular conduction delay (NICD) who possess a wide QRS without the appearance of left or right bundle branch block is also sparse. This ECG abnormality is present in between 3.8 and 5.8% of patients with impaired LV function [22, 23] and can be caused by a variety of pathophysiological processes, which may independently influence response. In the context of ischaemic heart disease, atypical electrical activation may occur around an area of necrotic tissue post myocardial infarction, modifying the appearance of a classic left bundle branch block morphology. Similarly, in peri-infract block, the trajectory of electrical activation becomes widened as it bypasses a previously infarcted area. Finally, NICD can occur in several cardiomyopathic processes as a result of increased LV myocardial mass and modifications in myofibrillar organisation.

Conflicting outcomes following the use of CRT in patients with NICD have been reported with some studies appearing to show benefits in quality of life [24], whilst others revealed no benefit in clinical composite score, remodelling or mortality [16, 17, 25, 26]. Morphology again appears to be the critical determinant with patients exhibiting a LBBB-like NICD morphology appearing to gain the most benefit. While reliably categorising the activation pattern on the basis of a 12-lead ECG alone can be challenging, this is possible using both invasive electroanatomical mapping or novel non-invasive body surface mapping technology.

#### Gender

**Fig. 4:**
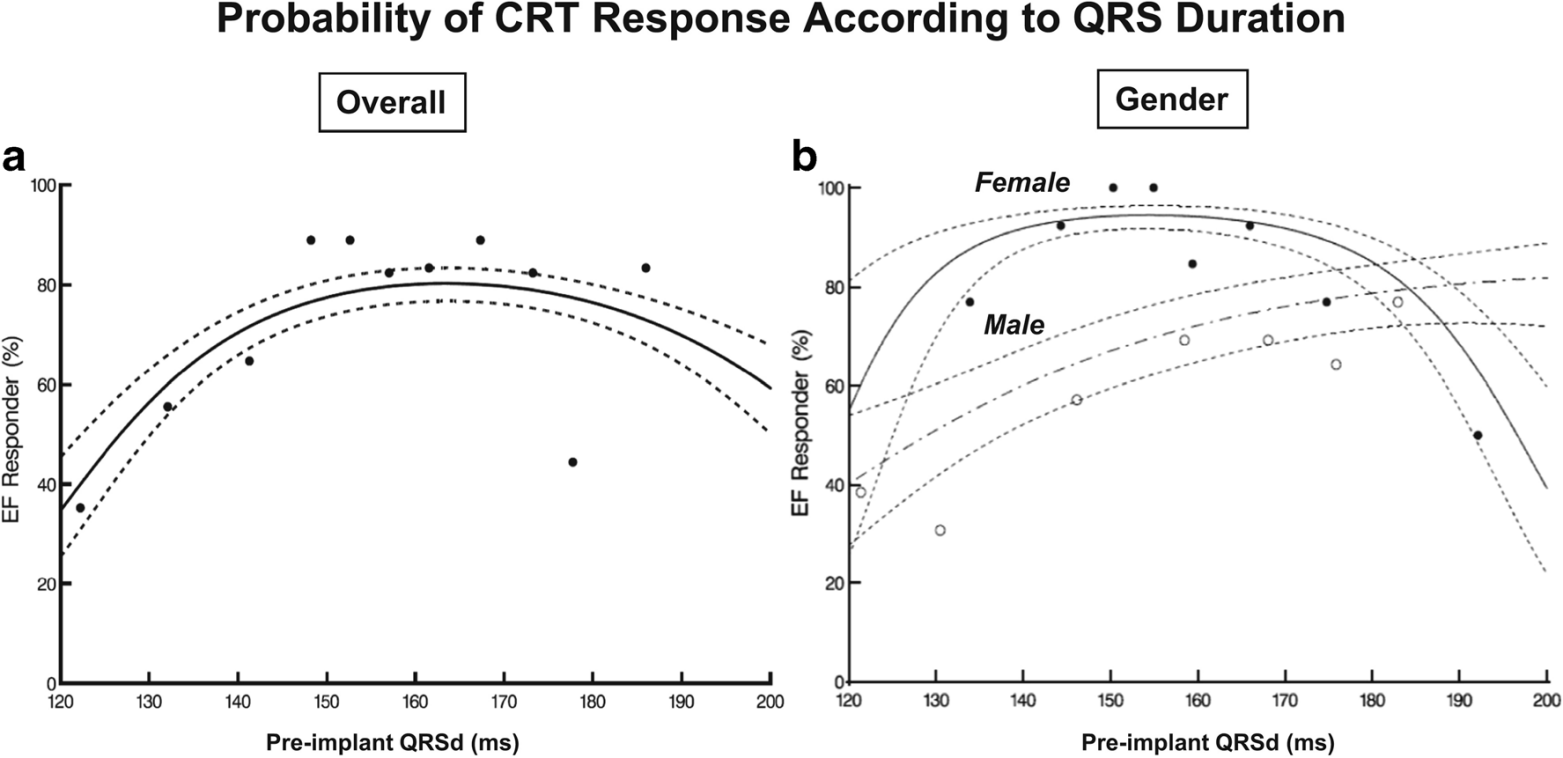
Probability of CRT response according to QRS duration (QRSd) as a continuous function. Parametric model: multivariable logistic regression shown with the corresponding 68% confidence limits (comparable to ± 1 SD). The decile points representing mean percentage of responders according to the deciles of QRSd are given as a crude verification of model fit. **a** Overall. Closed symbols represent decile points based on the equal number of patients (17 or 18 patients). **b** Gender-specific plot is based on a patient with baseline LVEDD 6 cm, baseline LVEF 20%, and 2 years from implant to follow- up echocardiography. Each decile point represents an average of ~ 10 patients (closed symbols: women; open symbols: men). Shapes were confirmed by semi- and nonparametric modelling. Reproduced with permission from [31]

Women have been consistently under-represented in nearly all large-scale trials of CRT and yet gender appears to play a key part in determining response to CRT [27–30]. Female CRT recipients appear to achieve superior survival benefits when compared to male recipients, although lower rates of ischaemic cardiomyopathy (ICM) confound this analysis [28, 30]. Interestingly, it appears that the degree of electrical dyssynchrony required to predict response to CRT differs between the sexes. The analysis by Varma et al. identified that the peak probability of response occurred with a comparatively narrower QRS than that of men [31]. Whilst the class 1 indication for CRT of a QRS > 150 ms affords men a ~60% chance of responding to CRT, women achieved the same level of response with a QRS of just 130 ms, see Fig. 4.

#### Aetiology and myocardial scar

In roughly 50% of patients receiving CRT, the aetiology of their heart failure will be ischaemic in origin [14]; however, ischaemic aetiology is an independent predictor of non-response to CRT [32, 33]. Other studies have shown that patients with ICM experience have less improvement in LVEF than patients with non-ischaemic, dilated cardiomyopathy (DCM) [34, 35]. The difference in remodelling has been attributed to a sequelae of the greater burden of scarred myocardium typically identified in ischaemic patients, reducing the potential for LV remodelling [36].

#### Atrial fibrillation

Around 25% of patients undergoing CRT implantation are in permanent atrial fibrillation (AF) [37]. It is common for patients with AF to concurrently exhibit older age, increased severity of heart failure and additional comorbidities than patients who present with sinus rhythm. Patients with AF are more likely to have a faster and more irregular heart than patients in sinus rhythm, and the aetiology of their LV dysfunction may in some instances be a result of tachycardiomyopathy. When the presenting rhythm was analysed in a randomised trial, CRT appeared to confer no benefit to patients with coexistent AF, whilst a 25% reduction in mortality was observed amongst those in sinus rhythm [38]. One explanation for this discrepancy is that amongst the group with AF, only 50% of the patients experienced a BiV pacing burden in excess of 90%. As such, the current European guidelines include the caveat that when contemplating CRT implantation in patients with AF, a class IIa indication exists, “provided that a BiV pacing as close to 100% as possible can be achieved” [10].

Given the importance of maximising the percentage of BiV pacing, it has been postulated that all patients with AF should undergo atrio-ventricular (AV) junctional ablation and this approach does appear to enhance the effects of CRT with the same magnitude as those seen in patients with sinus rhythm [39].

#### Upgrades to CRT

Patients with a bradycardia pacing system in situ who go on to develop heart failure symptoms account for around 23–28% of CRT implants. The deleterious effects of chronic RV pacing have been well established [40–42] and recently reviewed [43]. As such, implantation of a BiV pacing system may be appropriate for patients with evidence of symptomatic heart failure and reduced ejection fraction who are expected to experience a high pacing burden. It is likely stricter adherence to professional practice guidelines identifying suitable patients to undergo an upgrade to a CRT system will improve the response rate to the therapy.

### Peri-implant

Peri-implant factors associated with non-response to CRT include utilising appropriate LV lead technology and optimisation of the LV lead stimulation site.

#### LV lead technology

Transvenous, epicardial CRT was traditionally performed by implanting a bipolar pacing lead in a tributary of the coronary sinus, facilitating depolarisation of the LV. Multisite pacing (MSP) where stimulation is delivered from two or more sites within the LV would intuitively appear preferable to single-site stimulation. In early pilot work, MSP was associated with greater reverse remodelling [44, 45]; however, in a larger, randomised study of prior non-responders to CRT, no clinical or echocardiographic benefit was observed [46].

MSP can also be delivered via multipoint pacing (MPP). This technique utilises quadripolar LV pacing leads which have four integrated pacing cathodes along the course of the lead, allowing greater customisation from any of the of the 10–17 MPP vectors available. The increased choice for the implanting physician means it is possible to programme around frequently occurring issues including high pacing thresholds–potentially caused by areas of scar- or phrenic nerve stimulation, where LV depolarisation causes diaphragmatic twitching. The use of MPP has been shown to yield greater improvements in acute haemodynamic response [47] and in a small, randomised study, greater improvements in overall response [48].

#### Stimulation site

The optimal LV pacing site displays large inter and intra-patient variability amongst patients with both DCM [49] and ICM [50–52]. Delivering stimulation from a more apical position has largely been shown to yield less favourable outcomes [53, 54], and this practice is not endorsed in current guidelines [10]. In general, there is a consensus that the lateral free wall represents the most favourable target for LV lead deployment, typically within the lateral or postero-lateral cardiac veins of the coronary sinus [55–58]. Unfortunately, the constraints of the coronary sinus anatomy mean it is occasionally impossible to even implant an LV lead, let alone target a specific site which displays desirable viability and latency [59].

### Post implant

Post implant factors associated with non-response to CRT include remote monitoring, frequency of biventricular pacing, device programming and optimisation.

#### Remote monitoring

Almost all modern cardiac implanted electronic devices (CIEDs) have the capability to allow remote device follow up. Large, randomised studies have consistently shown that remote device monitoring is both feasible and reduces the need for ambulatory outpatient clinic attendance [60]. The use of remote monitoring has been shown to improve clinical outcomes for patients with heart failure as well as achieving a significant reduction in mortality [61]. However, similar benefits were not observed when a more rationalised approach to remote monitoring was adopted in other work [62].

Whilst CIEDs can analyse fluid status by calculating thoracic impedance as part of a multiparametric assessment, monitoring-only implantable technologies have also been devised. The CardioMEMS Heart Failure system (Abbott Medical Inc., Atlanta, GA, USA) is a wireless pulmonary artery haemodynamic monitor, which provides an accurate assessment of real time pulmonary artery (PA) pressure, allowing the physician to optimise pharmacotherapy. Use of this system was associated with a 33% reduction in hospitalisations when compared to standard of care [63]. Such systems appear to hold a great deal of promise, particularly if they could be integrated with the currently available CIED multiparametric assessments.

#### Biventricular pacing burden

In order for a CRT system to function effectively, it is essential that it is able to deliver consistent biventricular pacing. Frequent ventricular ectopics and atrial tachyarrhythmias can reduce the frequency of biventricular pacing and were identified in up to a third of non-responders to CRT [64]. There are several mechanisms by which atrial tachyarrhythmias reduce response to CRT. Rapid atrial rates can result in loss of ventricular stimulation, but of equal significance are the detrimental haemodynamic sequelae of the irregularity of the rhythm as well as the loss of intrinsic atrial function. A strategy of attempting to maintain sinus rhythm using pharmacological therapy in patients with heart failure conferred no benefit over a strategy of rate control [65]. Other strategies to increase the frequency of biventricular pacing include both catheter ablation [66, 67]. Recent work has suggested that this approach may even confer a survival benefit in patients with AF and heart failure [68]. AV junction ablation is an alternative strategy in patients with AF and whilst rendering the patient pacing dependant, appears highly effective when combined with biventricular pacing. As such, this practice is endorsed in the most recent guidelines [10].

Ventricular extra systolic beats can similarly disrupt the efficient function of a CRT system, reducing the frequency of effective biventricular pacing and as such, contributing to non-response. Again, the therapeutic target is to minimise the occurrence of such events either through the use of pharmacological therapy or catheter ablation [69].

#### Programming and optimisation

Appropriate programming can increase the frequency of biventricular pacing but is also essential in order to ensure the optimal mechanical functioning of the heart, which facilitates greater response. Arguably, the most important settings requiring optimisation are the pacing mode, upper and lower rate limits, the LV capture voltage, the stimulation vector and A-V and V-V intervals. Programming a high upper tracking rate ensures biventricular pacing is maintained during exercise. Similarly, the LV pacing output should include an adequate safety margin to ensure continual BiV pacing, although some modern devices can automatically adjust this parameter.

Iterative optimisation of the A-V and V-V intervals using doppler echocardiography is the established reference method of achieving optimal programming by ensuring optimal diastolic filling of the LV [70, 71]. Recent large multicentre studies evaluating this practice have shown it to be largely ineffective when compared to the use of empirical programming [72, 73]. A more promising technique, which optimises the A-V and V-V intervals using an integrated haemodynamic sensor, is currently undergoing further assessment [74].

## Biventricular endocardial pacing

The persistent rate of non-response to transvenous, epicardial CRT has led to the development of novel forms of undertaking CRT. Of these, biventricular endocardial pacing (BiV ENDO), where LV stimulation occurs from within the LV cavity, holds a great deal of promise. This technique is associated with several advantages over epicardial activation. BiV ENDO pacing can be achieved using several different approaches including the use of novel, leadless pacing.

### Benefits of biventricular endocardial pacing

A large body of evidence exists highlighting the potential benefits of BiV ENDO CRT.

#### Access to sites

Failure to implant an LV lead in a tributary of the coronary sinus during a transvenous, epicardial CRT procedure occurs in around 5–15% of cases [55, 75, 76]. Improvements in delivery kit [77] in conjunction with greater operator experience and the widespread adoption of quadripolar pacing leads [78] mean that in a more recent series, failure to implant an LV lead now occurs in under 5% of cases [79]. This can occur due to difficulty cannulating the coronary sinus ostium or passing the LV lead into a CS branch, unsatisfactory pacing parameters or phrenic nerve stimulation associated with LV pacing. Where LV lead implantation is feasible, it is confined to the available anatomy of the CS. Consequently, the final lead position is dependent on the presence of a suitable target vein and when none exists, it may be necessary to accept a sub-optimal position. In addition, around 26% of patients undergoing an upgrade procedure from a pre-existing bradycardia pacing system may have central venous stenoses preventing the implantation of a transvenous LV lead [80]. In this population, it can prove impossible to implant an LV lead in 20–50% of patients [81, 82].

BiV ENDO pacing is not reliant on the CS anatomy and instead, operators can choose to deliver stimulation at any site within the LV cavity. This allows for customisation of the stimulation site based on the patient’s pathology and physiology. In addition, whilst lead-based pacing systems may still encounter issues such as central venous stenosis and occlusion, particularly in the upgrade population, newer wireless pacing systems which can be delivered via a retrograde aortic approach eliminate the need for central venous access [83].

#### Phrenic nerve stimulation and activation threshold

Clinically relevant phrenic nerve stimulation (PNS) can complicate LV lead deployment during transvenous, epicardial CRT implantation and this complication is encountered in over 20% of patients at implant or at follow up [84]. Unfortunately, the likelihood of encountering PNS increases when the lead is deployed at sites most associated LV reverse remodelling. Reducing the LV pacing output is a recognised technique for overcoming PNS and it is important to note that endocardial capture thresholds appear lower than equivalent parameters observed during BiV EPI [85]. In addition, if PNS is encountered during implantation of a BiV ENDO pacing system, the operator has the freedom of the entire LV cavity to select an alternative pacing site.

#### Activation velocity

Endocardial stimulation of the LV facilitates more rapid myocardial depolarisation as wave front propagation occurs along the shorter endocardial surface of the heart. Several studies have also demonstrated enhanced conduction velocities associated with stimulation of endocardial tissue [86–88]. One hypothesis for this more rapid activation is the earlier recruitment of fast conducting Purkinje fibres with the capability of quickly disseminating activation. However, Purkinje fibres are largely electrically isolated from the surrounding myocardial tissue and require direct fibre stimulation [89]. Additionally, given the Purkinje fibres form a loose network within the LV endocardium, other factors are thought to play a more central role.

Hyde et al. set out to establish a greater understanding of fast endocardial conduction (FEC). Non-Purkinje sub-endocardial tissue conducts impulses faster than either mid-myocardial or epicardial fibres [89]. Instead, fibre orientation and anisotropy appear critical [87, 90] in addition to higher gap junction [91] and sodium channel [92] density and increased cell area at the endocardium [93].

#### Arrhythmogenesis

Traditional CRT delivers electrical stimulation to the epicardial surface of the LV wall. This pattern of activation is juxtaposed to the sequence exhibited during intrinsic myocardial activation which follows an endocardial to epicardial course. Reversing the normal depolarisation sequence is thought to promote repolarisation heterogeneity, which can prolong the QTc interval in susceptible patients and result in malignant arrhythmias [94–97]. In contrast, BiV ENDO pacing was associated with a reductions in both QT dispersion and T peak to end, two important markers of dispersion of repolarisation [98].

### Improving response through endocardial pacing

Increasing evidence supports the hypothesis that BiV ENDO pacing has the capability to achieve greater electrical resynchronisation and consequently, maximise response to CRT. Traditionally, BiV ENDO pacing has been performed in a relatively small number of patients, typically those with failure to achieve conventional CRT or those that have been non-responders. The recently published ALSYNC study was the first multicentre study of LV endocardial pacing and demonstrated the feasibility and safety of LV endocardial CRT delivered through the atrial trans-septal approach [99]. In 138 patients with either prior sub-optimal response to conventional CRT, failure of LV lead implantation or sub-optimal coronary venous anatomy, the investigators achieved a high implant success rate (89.4%), with stable pacing parameters and an 82.2% freedom from complications at 6 months. Furthermore, clinical and echocardiographic improvement was 59 and 55% respectively in a group with prior non-response, suggesting that LV endocardial pacing may overcome the issue of poor response to epicardial CRT. These figures are reinforced by a large meta-analysis of BiV ENDO which confirmed a meta-analytical echocardiographic response rate of 63.3% [100].

The acute haemodynamic benefits of BiV ENDO appear superior to those achievable utilising BiV EPI, even amongst a cohort of ischaemic, non-responders to conventional epicardial CRT [101]. When a direct comparison between BiV ENDO and BiV EPI was performed, the optimal endocardial location appeared superior to the optimal epicardial pacing site. However, the location of the optimal site was different in each patient and a sub-optimal endocardial location could be in fact prove detrimental to cardiac function. As such, if endocardial CRT is to be widely adopted, it is likely that tailoring the LV lead location to the individual patient’s anatomy, physiology and pathology will be required.

## Biventricular endocardial pacing systems

### Lead-based pacing systems

Chronic BiV ENDO CRT has traditionally been delivered via a pacing lead delivered via either a transventricular-septal or atrial-septal puncture. Due to the risk of thrombus formation resulting in systemic embolization, lead-based LV endocardial pacing requires lifelong anti-coagulation. In a recent meta-analysis, the stroke rate was found to be 2.5 per 100 patient years [100]. Often, these events were attributed to periods of reduced anti-coagulation given the difficulty associated with maintaining a therapeutic INR.

Following a trans-septal implant, the potential also exists for an adverse interaction between the mitral valve and an LV lead which passes through it. The presence of a pacing lead can worsen valvular regurgitation and this phenomenon has been acknowledged with right-sided leads. Reassuringly in the largest series of trans-septal LV endocardial pacing to date, no system-related valvular complications were recorded. Instead, echocardiography showed reductions in mitral regurgitation as a result of improved LV function [99]. The other major concern is the increased risk of mitral valve endocarditis should the system become infected. Secondary infectious foci including both cerebral and renal abscess formation are associated with the systemic embolization of vegetations.

#### Atrial trans-septal

Lead-based BiV ENDO is predominantly delivered via the atrial trans-septal approach [102]. Superior and inferior access is frequently required in order to puncture the atrial septum and successfully position the lead in the LV although several refinements of this procedure have been described.

#### Ventricular trans-septal

Ventricular trans-septal techniques allow the deployment of the lead using only superior access, eliminating the need for femoral punctures [103]. This approach may be associated with both a lower risk of stroke and disruption to mitral valve function by eliminating the presence of pacing leads in the left atrium crossing the mitral valve, although a target INR of 2.5–3 is still recommended [103].

#### Apical trans-septal

A hybrid surgical/percutaneous approach has also been described where the LV apex is accessed via a mini-thoracotomy facilitating the deployment of a lead at the LV apex, which is then tunnelled up to a generator, implanted in the conventional sub-clavicular position [104]. Whilst the results from this series of 20 patients appear promising, this is a more invasive option and a higher target INR of 3–3.5 is recommended.

### Leadless pacing systems

Whilst chronic BiV ENDO delivered via a pacing lead currently requires ongoing anti-coagulants due to the risk of thrombus formation, newer wireless technology almost entirely eliminated this risk. Formal anti-coagulation is not required and this development increases the likelihood of endocardial pacing becoming more widespread.

#### Transarterial leadless LV endocardial pacing

**Fig. 5:**
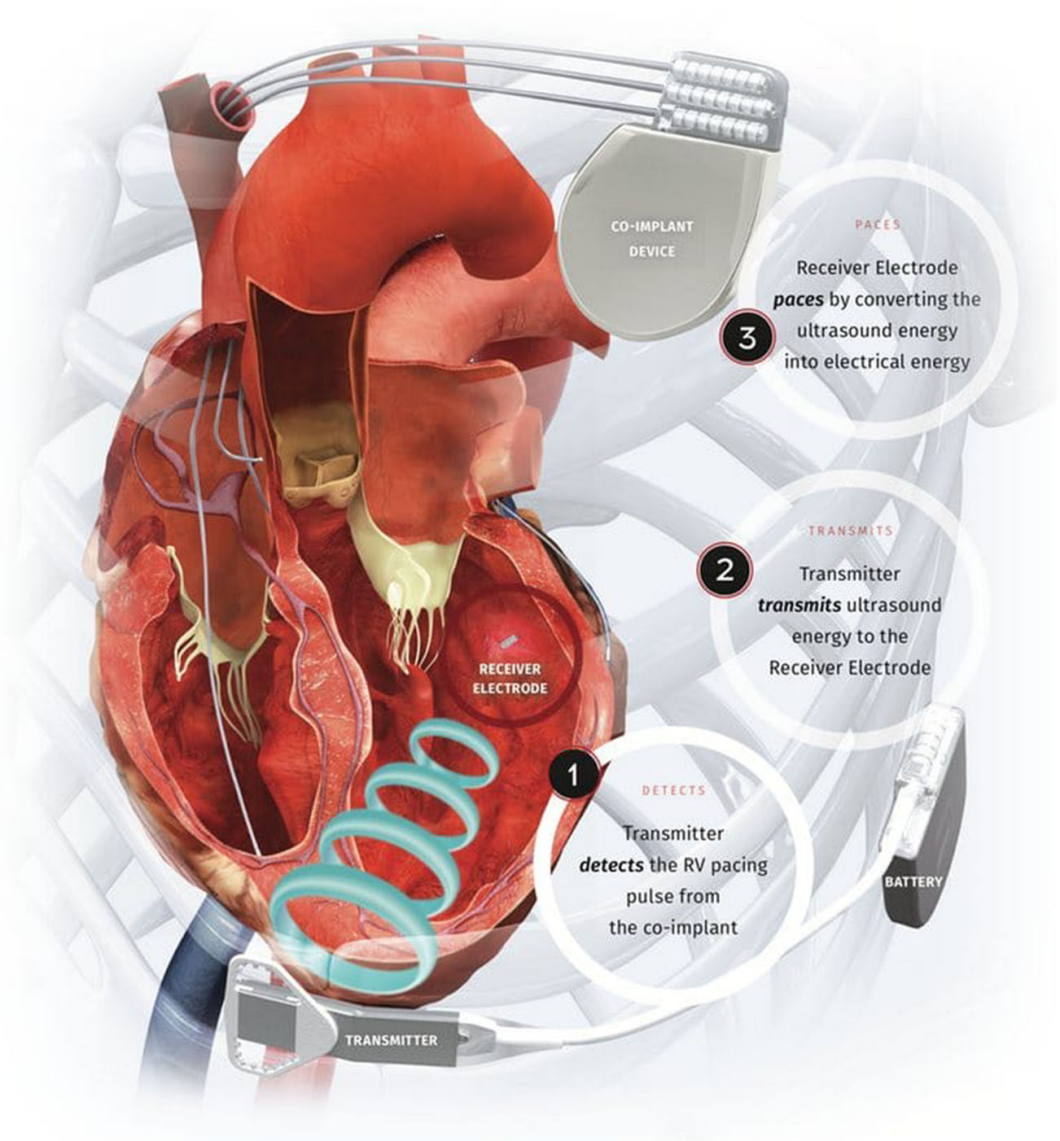
The WiSE CRT wireless biventricular endocardial pacing system. Reproduced with permission from EBR Systems, Sunnyvale, California, USA

A leadless BiV ENDO CRT pacing system has been developed, which eliminates the need for any form of pacing lead to be present within the left ventricle. Instead stimulation is delivered to the endocardial surface of the heart acoustically from an ultrasound (USS) pulse generator, implanted subcutaneously in an intercostal space, see Fig. 5. The USS waves stimulate a small receiver-electrode which is deployed percutaneously via the femoral artery, into the LV cavity. Acoustic USS energy is converted to an electronic pacing pulse by the receiver electrode. The pulse generator is triggered by RV pacing, resulting in near simultaneous (within 3 ms) LV and RV endocardial activation.

Whilst co-axial alignment of the USS pulse generator and pacing electrode is desirable, the USS transmitter uses beam-forming to direct the energy towards the pacing electrode, minimising the amount of energy required. Refinements of this generator mean it can now be implanted via a small horizontal incision and is attached to a dedicated battery, typically placed in the mid-axillary line via traditional lead tunnelling techniques. Battery life of the current system is typically quoted at 3 years, but newer battery technology currently nearing release is expected to double this longevity to over 6 years.

Early clinical studies evaluating this system confirmed implantation was safe and capable of achieving consistent biventricular capture [83] whilst more recent work has shown the system to be highly efficacious and associated with a clinical response rate of 84.8% [105].

#### Transvenous leadless LV endocardial pacing

The most commonly encountered complication associated with the use of the WiSE CRT system is femoral arterial access complications. In addition, not every patient has suitable anatomy to allow sufficient arterial access to allow the insertion of the electrode delivery catheter. Ischaemic heart disease is common in this population and as such, concurrent peripheral vascular disease is frequently encountered.

A method of transvenously accessing the LV cavity via an atrial trans-septal puncture has been described to facilitate deployment of the pacing electrode [106]. WiSE CRT implantation is commonly performed by electrophysiologists who are typically more comfortable obtaining trans-septal access than managing wide bore arterial access and as such, this route holds a great deal of promise. Early series evaluating its use have confirmed the safety of this approach [107].

## Conclusion

Non-response to CRT is a multifactorial issue. Improving patient selection and post implant device troubleshooting remain the cornerstone of optimising patient outcomes. Whilst newer lead technology including multipoint pacing and a greater focus on the importance of site selection are likely to be beneficial, the limitations of transvenous, epicardial CRT are widely acknowledged. In patients where implanting a transvenous lead is not possible, consideration should be given to endocardial pacing. Newer leadless LV endocardial systems no longer mandate lifelong anti-coagulation. In addition, endocardial pacing appears capable of yielding more effective electromechanical resynchronisation. Given the ability to stimulate the LV from any location, this technique would appear to offer a significant benefit to patients where transvenous lead implantation is restricted to inferior sites.

## Abbreviations

AHR: Acute haemodynamic response
CMR: Cardiac MRI
CRT: Cardiac resynchronisation therapy
CS: Coronary sinus
ICM: Ischaemic cardiomyopathy
LBBB: Left bundle branch block
LV: Left ventricle
LVEF: Left ventricular ejection fraction
LV_ENDO_: Left ventricular endocardial
NICM: Non-ischaemic cardiomyopathy
QLV: The interval between the onset of the QRS complex on the surface ECG to the first large positive or negative peak of the LV electrogram during a cardiac cycle
QRSd: QRS duration
RV: Right ventricle
